# Quantification of heterogeneity observed in medical images

**DOI:** 10.1186/1471-2342-13-7

**Published:** 2013-03-02

**Authors:** Frank J Brooks, Perry W Grigsby

**Affiliations:** 1 Department of Radiation Oncology, Washington University School of Medicine, 4921 Parkview Place, Saint Louis MO 63110, USA; 2 Division of Nuclear Medicine, Mallinckrodt Institute of Radiology, Medical Center, Saint Louis MO, USA; 3 Department of Obstetrics and Gynecology, Washington University Medical Center, Saint Louis MO, USA; 4 Alvin J. Siteman Cancer Center, Washington University Medical Center, Saint Louis MO, USA

## Abstract

**Background:**

There has been much recent interest in the quantification of visually evident heterogeneity within functional grayscale medical images, such as those obtained via magnetic resonance or positron emission tomography. In the case of images of cancerous tumors, variations in grayscale intensity imply variations in crucial tumor biology. Despite these considerable clinical implications, there is as yet no standardized method for measuring the heterogeneity observed via these imaging modalities.

**Methods:**

In this work, we motivate and derive a statistical measure of image heterogeneity. This statistic measures the distance-dependent average deviation from the smoothest intensity gradation feasible. We show how this statistic may be used to automatically rank images of *in vivo* human tumors in order of increasing heterogeneity. We test this method against the current practice of ranking images via expert visual inspection.

**Results:**

We find that this statistic provides a means of heterogeneity quantification beyond that given by other statistics traditionally used for the same purpose. We demonstrate the effect of tumor shape upon our ranking method and find the method applicable to a wide variety of clinically relevant tumor images. We find that the automated heterogeneity rankings agree very closely with those performed visually by experts.

**Conclusions:**

These results indicate that our automated method may be used reliably to rank, in order of increasing heterogeneity, tumor images whether or not object shape is considered to contribute to that heterogeneity. Automated heterogeneity ranking yields objective results which are more consistent than visual rankings. Reducing variability in image interpretation will enable more researchers to better study potential clinical implications of observed tumor heterogeneity.

## Background

There are hundreds of papers in the medical literature about the importance of heterogeneity within various types and degrees of cancerous tumors. In oncology parlance, “tumor heterogeneity” generally means that a whole tumor comprises distinct cellular sub-populations [1]. These cell groups vary in morphology, histology and growth rate, for example. The interactions of different tumor cells with each other and with the microenvironment are complex and not well understood [2]. In order to understand intra-tumor biology, potentially subtle differences near and within tumors must be quantified. The ultimate goal of tumor heterogeneity studies is to determine the implications and prognostic value of observed variations in a host of clinically relevant tumor properties such as physical size, shape, cellular density, cellular metabolism, hypoxia and vascularization. Each of these biologically interesting properties generally are assayed via an imaging modality—such as magnetic resonance (MR), computed tomography (CT) or positron emission tomography (PET)—which outputs grayscale images. Thus, the challenge of measuring biological heterogeneity manifests as the quantification of visually evident intensity variations in grayscale images.

**Figure 1:**
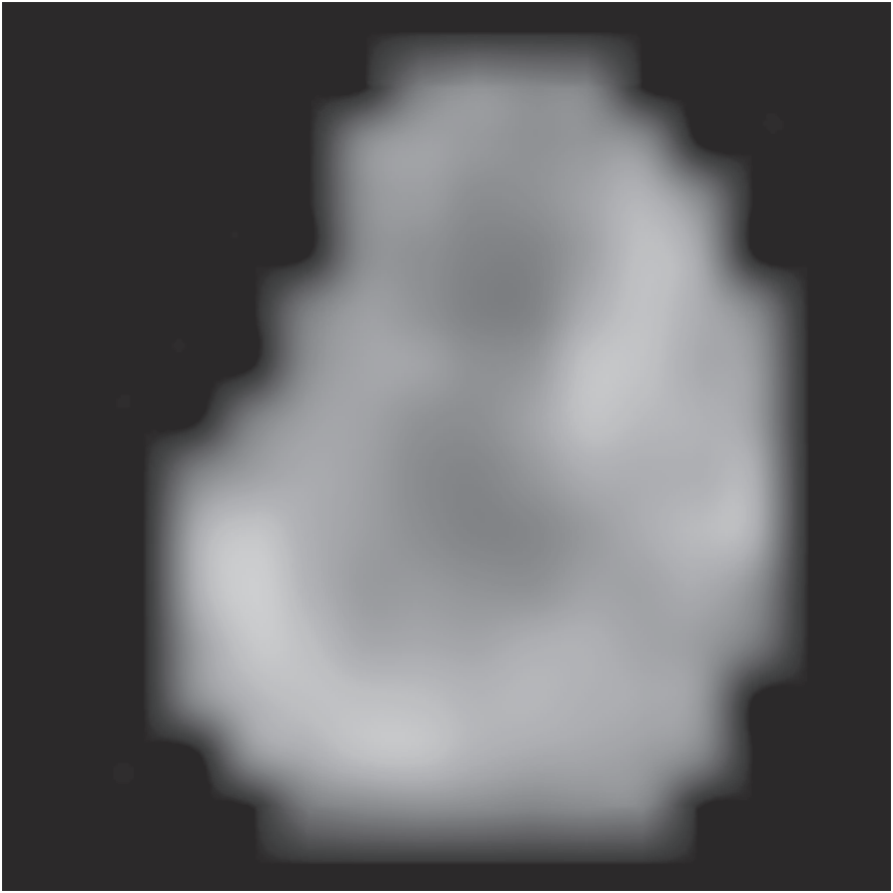
**Variations in the grayscale intensity of this tumor are evident.** For example, contrast the bright region at the bottom-left to the darker region in the center. The regions of varying intensity are amorphous an diffusely bounded. In the present case, these regions represent the heterogeneous uptake of a radio-labeled glucose analogue (FDG). The vertical edge of this image corresponds to 64 mm. This image is also seen in Figure 4 as panel F1.

Consider, for example, the PET image of a cancerous tumor shown in Figure 1. Within the bulk of the tumor, variation in the grayscale pixel intensities is seen. Toward the bottom-left, a region of the brightest pixels is seen to smoothly gradate to darker regions. Similar distinct darker regions then are seen at the center and toward the top of the bulk, although neither follow an easily described shape or pattern. Similarly amorphous bright regions punctuate the periphery of the tumor bulk. It is precisely these variations which interest the researchers because variation in PET intensity implies variation in biological properties of the tumor. When one attempts to perform a clinical study of those biological properties via PET image data, two significant obstacles arise. First, the variation in observed pixel intensity is difficult to describe verbally [3]. As such, there is no convention for describing the properties seen in tumor images. Thus, even experienced clinicians are unlikely to describe the same tumor in the same manner or, much less, even agree upon which image features are salient. This complicates the development of statistics used to quantify “obvious” intensity variations. Second, the implementation of any statistic will have to contend with relative paucities of pixel data. Consider, for example, that a typical PET scanner renders a 4×4×5 mm^3^ physical region as a single pixel in the final image [4]. If one assumes that a clinically typical tumor volume of 40 cm^3^ is a sphere, then the largest cross-sectional image contains only ≈90 tumor pixels. In fact, it is common that oncology studies include a large fraction of PET images where fewer than 20 pixels comprise the entire tumor region. Therefore measuring the variation within these small regions can be difficult since the use of many statistics—including those common to texture analysis and correlation techniques [5]—requires large sample sizes.

Several attempts to objectively quantify tumor heterogeneity have been published previously [6-10]. These attempts largely have yielded mixed results. The more rudimentary measures are non-spatial. That is, they depend only upon the distribution of intensities, not the spatial arrangement of those intensities within the tumor. For example, although the standard deviation, skewness and kurtosis clearly quantify variation of an intensity distribution, skewness and kurtosis have been shown not to vary sufficiently to separate some cervical cancers into groups of differing disease outcome [10]. Another non-spatial measure of heterogeneity which was shown to distinguish patient groups [6] was later demonstrated to actually be a surrogate for tumor volume [10]. It has been claimed that the image texture metrics introduced in the seminal work of Haralick et al. [11] can be used as spatial measures of heterogeneity [8,9]. However, many of those more sophisticated metrics have been shown later to be statistically irreproducible on tumor data [12]. In general, even in cases where enough image data exists to yield consistent inter-image comparison, it remains unclear which texture metric corresponds to which subjectively perceived image quality [11]. In short, there is no standard for measuring tumor heterogeneity in a manner consistent with expert visual inspection.

In this study, we develop and test a novel statistic which may be used to quantify spatial variations in tumors imaged *in vivo* which were identified and segmented via independent analysis. In other words, we quantify the spatial heterogeneity within definitively bounded objects against a uniform background. Here, we do not seek to attach any clinical or prognostic meaning whatsoever to a given image or statistical value. Instead, we seek to give the clinician a quantitative mechanism of declaring one image to be *x* times more heterogeneous than another, such that a set of similarly imaged objects may be ranked and compared objectively. We compute our statistic on real PET images of tumors exhibiting visually manifest variations in size, shape and intra-tumoral intensity and find that our numerical heterogeneity comparisons agree well with the subjective comparisons made by experienced experts.

## Methods

### Motivation

Heterogeneity has application-specific connotation. Common to all applications, however, is that heterogeneity is necessarily defined by the scale of interest. For example, at scales much smaller than a single square, a chessboard image could be interpreted as homogeneous. This is due to the fact that at very small scales, neighboring pixels are much more likely to be in the same square than they are in one of differing color. At a scale on the same order of magnitude as the squares, the chessboard is more heterogenous because any pixel about one square size away from an arbitrary pixel has approximately equal chance of being either color. This is greater average variation than in the previous case of usually being confined to a single square. If the scale of interest becomes much larger than the size of a single square, the image may again be considered homogeneous since, on average, anywhere in the image, the pattern is precisely the same. When attempting to quantify the heterogeneity in an image, one must make clear the intended scale of interest.

Contrast the scale-dependence of heterogeneity to the definitiveness of homogeneity. Ultimate homogeneity is unambiguously defined as the state being the same at all scales, e.g., an image of only a single color. We therefore assume that heterogeneity may be clearly defined as difference from homogeneity. Of course, a perfectly homogeneous image contains no information and is therefore of little practical use. The real images we study will contain pixels of varying grayscale intensity. We thus seek to first find the most homogeneous intensity transition between given pixels with differing intensities. Consider two distinct image pixels where the grayscale intensity is *I*_1_ at pixel one and *I*_2_ at pixel two. Assume that *I*_1_>*I*_2_. Let *r* be the distance measured from pixel one to an arbitrary point along the straight line segment between pixels one and two. The gradation between pixels one and two which is as locally homogeneous as possible, for every distance *r*, occurs when the whole intensity change is spread evenly across the entire line, *i.e,* when the derivative *dI*/*dr* is constant. We consider any deviation from that smoothest possible transition to be heterogeneity.

### Mathematical development

We are interested in measuring the variation within grayscale image objects. We assume these objects have already been identified and isolated via relevant standards. We therefore begin with a definitively bounded object against a uniform background of zero intensity. We take as input a pair of distinct object pixels, which we arbitrarily label as *m* and *n*. The *m*^th^ pixel at image coordinate (*x*_*m*_,*y*_*m*_) has a grayscale intensity *I*_*m*_. The *n*^th^ pixel at image coordinate (*x*_*n*_,*y*_*n*_) has a grayscale intensity *I*_*n*_. The pair separation distance *r*_*mn*_ is simply the Euclidean distance between the *m*^th^ and *n*^th^ pixel. The inter-pixel intensity change is *I*_*mn*_=*I*_*n*_−*I*_*m*_. The minimal discrete set of pixels connecting the *m*^th^ and *n*^th^ pixels is taken to be the Bresenham line [13] between those pixels, to which we collectively refer as ℒ. Thus, ℒ is an ordered set of pixels comprising the straightest line that can be drawn from the *m*^th^ pixel to the *n*^th^ pixel while still being constrained to a discrete lattice of non-fractional pixels. The smoothest possible gradation between these endpoints occurs when *I*_*n*_ monotonically decreases (or increases) to *I*_*m*_ over ℒ exhaustively. That is, when the intensity change is spread evenly across the entire line connecting the endpoints. We compute the grayscale value yielding the most homogeneous gradation

(1) $$I(r_{\text{ml}})=I_{m}+\frac{I_{n}-I_{m}}{r_{\text{mn}}}r_{\text{ml}}$$

where *r*_*ml*_ is the Euclidean distance between the *m*^th^ pixel and *l*^th^ pixel in ℒ. The absolute difference between the homogenous grayscale value *I*(*r*_*ml*_) and the actual value at the *l*^th^ pixel *I*_*l*_ is computed for each pixel in ℒ. These differences are summed over ℒ and then divided by the discrete pair separation *L*, *i.e.,* by the number of elements in ℒ. We thus have computed

(2) $$\bar{\mathrm{\Delta I}}=\frac{1}{L}\underset{l\in ℒ}{\sum }|I(r_{\text{ml}})-I_{l}|,$$

the average absolute intensity difference along a line connecting a pair of object pixels.

For each non-repeating pair of object pixels, we compute the discrete distance *L* and $\bar{\mathrm{\Delta I}}$. The result is a pool of values where any one value of *L* has associated with it many values of $\bar{\mathrm{\Delta I}}$. Those multiple $\bar{\mathrm{\Delta I}}$ values are then ensemble-averaged for each *L* such that each discrete pair separation possible then has associated with it only a single value, $\bar{\bar{\mathrm{\Delta I}}}\equiv {\langle \bar{\mathrm{\Delta I}}\rangle }_{\text{ens}}$. The discrete distances are normalized to the largest discrete distance ( $\tilde{L}$) observed amongst any object pixel pair. The $\bar{\bar{\mathrm{\Delta I}}}$ are then plotted versus $L/\tilde{L}$.

The resulting plot may be interpreted as follows. In essence, the plot measures how the average deviations from homogeneity scale with percent object size. As that percent distance approaches unity, the opportunity to accrue larger deviations from homogeneity increases. The simplest assumption is that object heterogeneity will then accrue proportionally as more of the object is considered. Thus, a curve observed to be well below the proportionality line implies that progressively greater spans across the object tend not to accumulate differences from a smooth gradation. In other words, that object tends to be more homogeneous. Conversely, an object with curve well-above the proportionality line tends to be more heterogenous since deviations from smoothness accrue even across smaller spans. One simple means of quantifying the qualitative observation of a curve being above or below the proportionality line is via the area under that curve. We therefore define our heterogeneity quantification statistic to be

(3) $$\zeta \equiv \int_{0}^{1}\bar{\bar{\mathrm{\Delta I}}}(L/\tilde{L})\;d(L/\tilde{L}).$$

Because the distances plotted are relative to the size of the object, objects of greatly varying size may be compared to one another. This enables the use of *ζ* as a ranking statistic where increased *ζ* implies increased heterogeneity.

### Implementation

Computer code to automatically compute *ζ* on grayscale images was written in Python v2.6.1 ( http://www.python.org). Input images consist of an 8-bit grayscale object, where each object pixel has intensity *I*>0, against a uniformly black background (*I*=0). The images were read into the program using the Python Imaging Library v1.17 ( http://www.pythonware.com/products/pil/). The *xy*-coordinate at each nonzero pixel (an object pixel) along with the intensity at that pixel was recorded. For every unique pixel pair, the discrete line between the pair was calculated using Bresenham’s algorithm [13]. Here, we do not consider the pairs directionally. Thus, once a particular line from pixel *m* to pixel *n* has been considered, we do not consider again the line from *n* to *m*. We take adjacent pixels to have zero separation and thus there can be no intensity difference from the gradation between them. For line endpoints separated by more than one pixel, the smoothest gradient possible was computed using Equation 1. For each pixel between the endpoints, the average absolute difference between the measured intensity and that given by Equation 1 was computed via Equation 2. These averages were binned together for each integer value of the discrete line length. These binned averages then were themselves averaged, resulting in a single value at each discrete line length ( $\bar{\bar{\mathrm{\Delta I}}}$). Each integer line length was divided by the maximum integer pair-separation observed for the image object. Using Equation 3, *ζ* was computed via the trapezoidal rule as implemented in Numpy v1.4 ( http://www.numpy.org).

### Test image database

#### Images used for numeric tests

Images were obtained as described in Ref. [10]. We briefly recapitulate the process here. Patients with cancers of the uterine cervix underwent a pre-treatment hybrid PET/CT scan using the ^18^F-fluorodeoxyglucose (FDG) radiotracer assay of glucose uptake by cells. As is common throughout the field of nuclear medicine, the raw FDG-PET data are scatter and attenuation corrected via the proprietary software native to the PET machine. Images were reconstructed using ordered subset expectation maximization. A Gaussian smoothing filter with 4-mm full width at half maximum was applied post-reconstruction. No additional processing was implemented. In order to objectively distinguish tumor from background, we employed the rule-of-thumb that, for a visually selected region of interest (ROI), any pixel brighter than 40% of the maximum ROI pixel brightness is to be considered part of the tumor [14]. Any remaining objects that are obviously (for sound anatomical reasons) not tumors are removed and the final ROI is exported as an 8-bit grayscale region against a uniformly black background. Here, variations in grayscale intensity ostensibly imply variations in intra-tumoral metabolic activity [4]. To quantify these variations, we apply the computer code earlier described to the largest cross-sectional tumor image for each patient.

#### Images used for visual tests

The whole-body images created by our PET scanner are only 168 ×168 pixels in size. The segmented tumor region within these images ranges from only 16 to 291 total pixels with a median of 63 pixels. These regions are generally too small to be inspected visually. Computer code using the Python Imaging Library (PIL) was used to automatically extract the pre-defined tumor region and paste that at the center of a new image such that the tumor is centered within a border which is half of the diameter of the tumor. This new image was expanded to 162 ×162 pixels using the resize function of the PIL with the filter option set to nearest-neighbor. At 72 dots per inch, this corresponds to squares of side length 2.25 inches. Each image was printed and then pasted onto a separate piece of stiff card stock such that a group of images could be readily seen at once. The expert can then manipulate the cards into order of increasing image heterogeneity, *i.e.,* visually evident intensity variations within an isolated tumor region.

In the rescaled images, very small tumor regions will appear manifestly “blockier” than very large ones. This is the typical pixelation seen whenever low-information images are expanded to greater than the original image size. The result is that a highly pixelated image may appear innately more homogeneous than a less-pixelated image simply due the relative sizes of the original pixels in the rescaled images. Furthermore, the blocky appearance of the rescaled tumor regions could give a clever physician some indication of tumor size, even without anatomical reference. Since we do not want the physicians to be biased by innate clinical knowledge regarding tumor size, we binned tumors of similar size into distinct groups which were independently inspected by the experts.

### Comparison of test results

The test image sets earlier described were given to: an experienced clinical oncologist, a senior medical resident in the oncology department and a professional image processing expert with no medical background. Each expert was asked to rank each image set individually in order of increasing heterogeneity. Only the experienced oncologist, however, considered tumor shape as a heterogeneity-defining quality. The other two experts specifically ignored tumor shape and focused only upon intra-tumoral pixel variations. The computer code earlier described was used to compute an objective ranking of each image set based solely upon increasing values of the *ζ* statistic. The *ζ*-ranking for each image set was compared to each expert ranking via the Spearman rank test of statistical correlation. As described in many textbooks, a *p*-value associated with an upper-tailed test of significance may be obtained from the Spearman correlation coefficient. Our null hypothesis is that the computer/expert rankings are totally uncorrelated. Our alternative hypothesis is that larger *ζ* values tend to pair with higher expert-ranked-heterogeneity.

### Test of shape dependence

To explore the dependence of *ζ* upon object shape, we performed the following test. An 8-bit grayscale image of a circle with radius 16 pixels and origin at the image center was created. The circle was shaded via a two-dimensional Gaussian distribution such that the brightest intensity (255) is at the circle center and the dimmest intensity (128) occurs at the circle circumference. The result is a smoothly varying, symmetric “tumor” object. The object was then decimated by setting all pixels to zero within circular holes of radius 4 pixels centered at random locations within the object. As decimation is performed repeatedly upon the same object, the shape of the object becomes increasingly irregular. To test the effect of shape upon the *ζ* statistic, we modified the calculation given in Equation 3 to consider contributions from only the nonzero pixels along the connecting Bresenham lines and replacing the pair-separation *L* in Equation 2 with the number of pixels considered. This way, if the line between a pair of object pixels crosses the background, the differences of the background pixels from the smoothest gradation are ignored. This effectively renders any two background-separated pixels to be neighbors, thus closing the shape while maintaining the relative orientation of one endpoint to the other.

## Results and discussion

**Figure 2:**
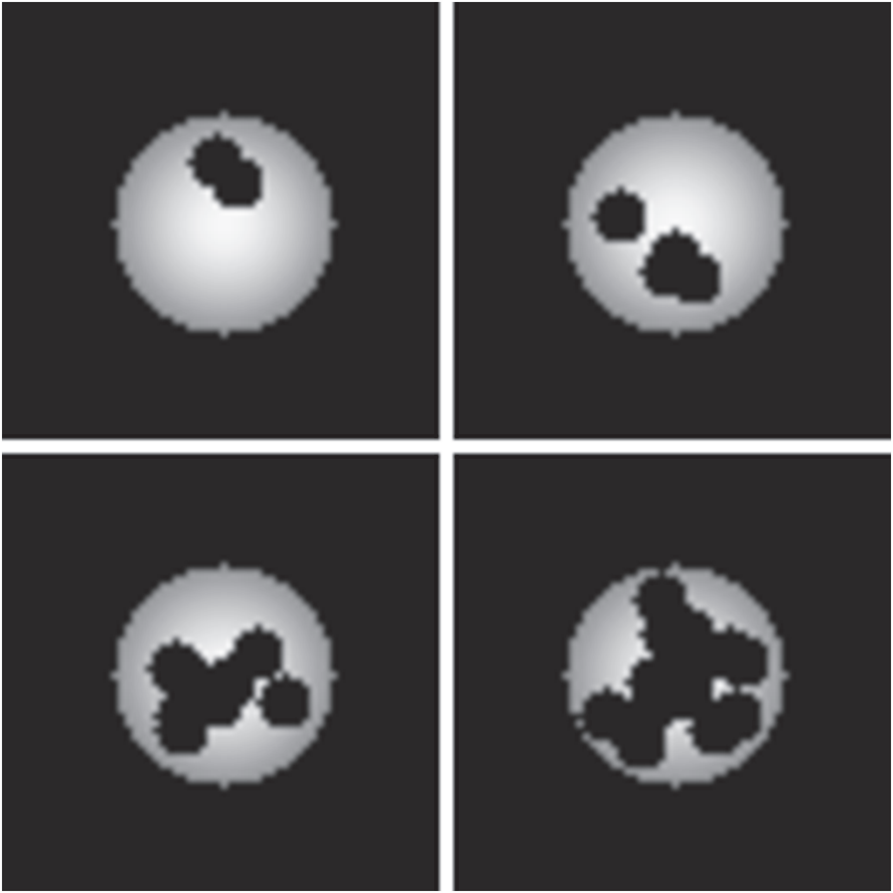
**Repeated random decimation of a symmetrically shaded circle was used to test the shape dependence of the *ζ *statistic.** Here, typical images of 2, 4, 8 and 16 decimations are shown. The images shown have been resized for display purposes.

**Figure 3:**
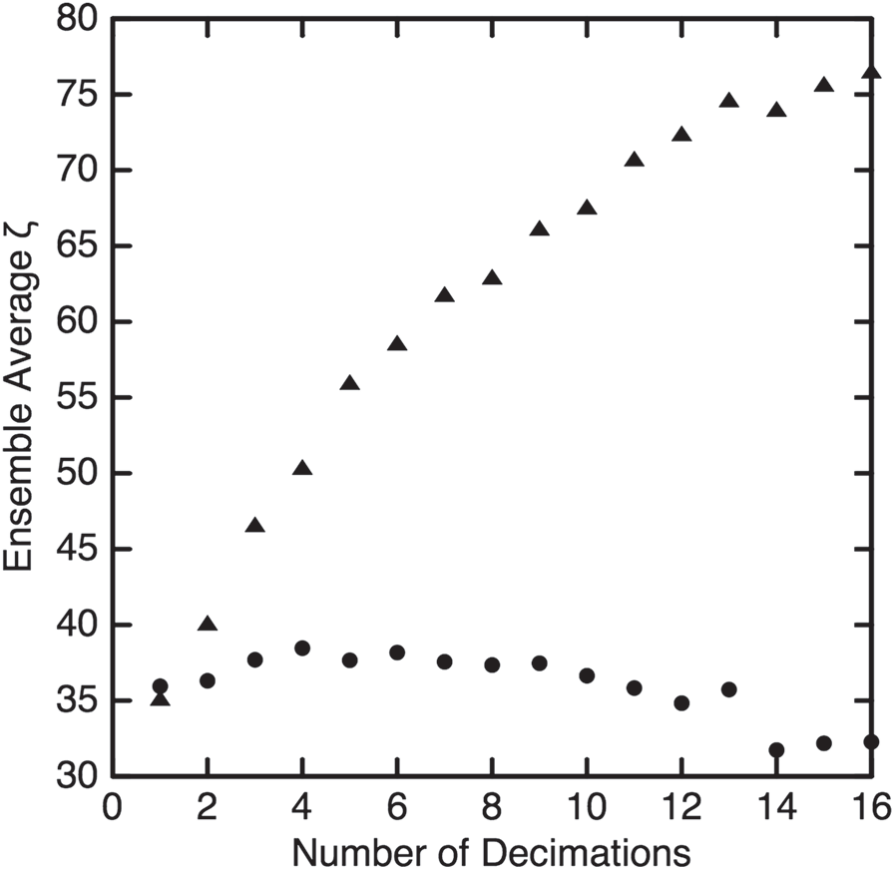
**The *ζ *statistic was calculated in shape-aware (triangles) and shape-unaware (circles) modes.** As the object becomes more irregularly shaped with repeated decimation, the shape-aware heterogeneity measure increases by a factor of two while the shape-unaware metric exhibits little variation.

Because only some oncologists consider shape to contribute to overall tumor heterogeneity, we first describe the effect of object shape upon *ζ*. As described in the Methods section, a smoothly shaded circle was randomly decimated with circular holes. For a given number of decimations, an ensemble of 36 images was created. Some typical examples are shown in Figure 2. The *ζ* statistic was calculated as given in Equation 3 for each image in the ensemble and the results averaged to yield a single “shape-aware” value for a given number of decimations. This process was then repeated for each ensemble using modification earlier described to yield a single “shape-unaware” value, *ζ*_*u*_, for a given number of decimations. The result is shown in Figure 3 where the contrast between *ζ* (triangles) and *ζ*_*u*_ (circles) is stark. We note that *ζ*_*u*_ varies only ≈5% from the average value and thus shows that *ζ*_*u*_ is effectively blind to the profound changes in object shape. In contrast, as the object becomes more irregular in shape, *ζ* increases by a factor of two. Additionally, because the greatly decimated objects have much less non-zero object area than do the slightly decimated objects, the lack of variation in *ζ*_*u*_ demonstrates independence from object size.

**Figure 4:**
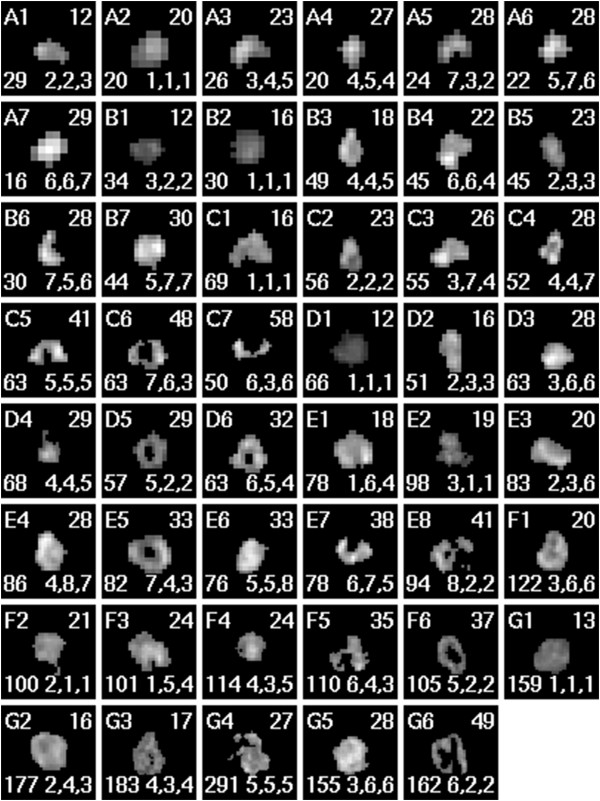
**Tumors of various size, shape, mean intensity and heterogeneity are shown as assayed via FDG-PET.** The letter-number at the top-left indicates the set and *ζ*-rank within that set. The number at the top-right is the rounded *ζ* value. The number at the bottom-left is the area of the tumor given in pixels. The sequence of numbers below is the ranks (within the single set) given to the image by the oncologist, the resident and the professional, respectively. The images above were rescaled via nearest-neighbor filtering to a convenient display size; thus, the sizes shown should not be compared directly.

The *ζ* statistic was computed for each of the images shown in Figure 4. Within each image subset (labeled A–G), the images were ranked automatically in order increasing *ζ* value. These are the rankings compared to that of the experts via the Spearman rank test. Table 1 shows the associated *p*-values which are the likelihood that a positive automated/expert rank correlation is due entirely to chance alone. As indicated by the low *p*-values in the third column, the automated rankings via *ζ* value agree very well with those given by the veteran oncologist. Such close agreement is expected to occur by chance in only 1 out of every 25 attempts. Contrast this to the rankings given by either the oncology resident or professional image processor, where similar agreement with the automated ranking is expected to occur in 1 out of every 5 attempts. It is thus seen that *ζ*-ranking agrees very well with those who consider object shape contributions to heterogeneity and does not agree with those who ignore object shape.

**Table 1:** Automated, shape-aware ranking versus ranking by human experts

		**Shape aware automated ranking**		
		**Shape aware**	**Shape unaware**	
**Set**	**Area(px)**	**Oncologist**	**Resident**	**Professional**
A	0–30	0.015	0.020	0.121
B	30–50	0.100	0.025	0.020
C	50–75	0.003	0.185	0.075
D	50–75	0.003	0.220	0.288
E	75–100	0.003	0.375	0.423
F	100-125	0.083	0.250	0.250
G	125–300	0.050	0.250	0.220
Mean		0.04±0.02	0.19±0.05	0.20±0.05

The correlation between the heterogeneity rankings computed via the shape-aware *ζ* statistic and those done by human experts was quantified via the Spearman rank test. The *p*-values given in columns 3–5 are the likelihood that observed correlations are due entirely to random chance. The agreement between the shape-aware automated ranking and the shape aware expert is outstanding.

This result was confirmed employing the modified, shape-unaware calculation described earlier. Our prediction is that *ζ*_*u*_ will agree with the two experts who ignored shape in their rankings while disagreeing with the expert who considered shape to be important. As seen in Table 2, this is precisely what occurred. The expected chance agreement between the automated and expert rankings is almost perfectly reversed from those seen in Table 1.

**Table 2:** Automated, shape-unware ranking versus ranking by human experts

		**Shape unaware automated ranking**		
		**Shape aware**	**Shape unaware**	
**Set**	**Area(px)**	**Oncologist**	**Resident**	**Professional**
A	0–30	0.038	0.010	0.038
B	30–50	0.100	0.015	0.020
C	50–75	0.389	0.075	0.050
D	50–75	0.067	0.025	0.010
E	75–100	0.200	0.100	0.021
F	100-125	0.250	0.130	0.083
G	125–300	0.160	0.100	0.123
Mean		0.17±0.05	0.06±0.02	0.05±0.02

Same as Table 1 except that automated ranking via *ζ*_*u*_ was implemented. As is seen in columns 4 and 5, the shape-unaware automated rankings agree very well with the two experts who did not consider shape as contributing to overall object heterogeneity.

We further investigated this sensitivity to shape as follows. Inspection of Figure 4 shows that images D5, D6, E5 and F6 each have the distinctive shape feature of a hole at the center. For these images of ring-like objects, 29≤*ζ*≤37. If one scans the entire image set, one finds five other images with a *ζ* value in this range. These images—such as B7, E6 and F5—are clearly not shaped similarly to the ring-like objects. It is thus seen that although the ring-like objects differ in *ζ* value by only 10% of their mean value, all *ζ* within this range do not correspond to similar objects. This demonstrates that while *ζ* is sensitive to object shape, it is not slaved to it. That is, one particular tumor shape does not uniquely correspond to a particular *ζ* value. This may be seen concisely in images D4–D6 where *ζ*_D4_=*ζ*_D5_, but have totally different shapes, while D5 and D6 have very similar shapes but *ζ* values which differ by about 10%.

Despite the ordered appearance of the rescaled images shown in Figure 4, clinically relevant tumor images vary greatly in size. This may be also seen in Tables 1 and 2 where the range of cross-sectional tumor sizes varies tenfold. Since it is the relative *arrangement* of pixel variations that ultimately defines heterogeneity, a robust quantifier of heterogeneity should not scale with image size. For example, doubling the size of a chessboard does not change the size of the squares, instead, there simply are more of them. Thus, the heterogeneity is the same for both sizes of chessboard. With this in mind, we explored the correlation between *ζ* and tumor size. Since the *ζ* values for the pool of images shown in Figure 4 do not follow a normal distribution, the traditional Pearson product moment is not applicable. We therefore employ the Spearman rank test of correlation and find *ρ*_*S*_=0.044 (*p*=0.39), which implies no appreciable correlation between *ζ* and object size. This is expected because the effective diameter of the object ( $\tilde{L}$) is divided out of Equation 3.

As mentioned in the Background, there have been mixed results from previously proposed heterogeneity metrics. We therefore tested *ζ* against the measures shown to be more reproducible and clinically applicable. Specifically, these metrics are the variance of the intensity distribution, the local entropy, image energy, image contrast and local homogeneity [8-10,12]. The latter four metrics are calculated from intensity co-occurrence matrices as described in Ref. [11]. In brief, a co-occurrence matrix describes the probability that pixels of differing shades occur as fixed-distance neighbors. As these matrices are innately directional, we computed the matrices first in the horizontal (angle=0) and then in the vertical (angle= *π*/2). A given heterogeneity metric was then computed from each matrix and root-mean-square averaged into a single measure. In this manner, we computed each of the proposed metrics on each of our test images. These values are given in Table 3. Ranking of each image set via each of the metrics (including *ζ*) was done and compared to those done independently by the veteran oncologist. As seen in Table 4, *ζ*-ranking is by far the most consistent with the human expert. In fact, the variance, local entropy and image energy rankings, on average, do not agree with expert analysis any better than random chance. The next best correlation is given by the image contrast which, on average, clearly did not perform as well as *ζ* in ranking our test images consistent with the veteran oncologist.

**Table 3:** **The values of the heterogeneity metrics are given for each of the images shown in Figure**4

**Image**	***ζ***	**Variance**	**Entropy**	**Energy**	**Contrast**	**Homogeneity**
A1	11.88	1637	3.525	0.03021	5587	0.02554
A2	20.14	2631	3.219	0.04000	4161	0.01220
A3	23.29	2653	3.438	0.03268	5920	0.01479
A4	26.94	2335	3.239	0.03924	6877	0.0009710
A5	27.57	2011	3.395	0.03395	5083	0.002498
A6	28.34	3126	3.332	0.03571	8388	0.01974
A7	29.40	5136	3.044	0.04762	10900	0.004394
B1	11.87	727.8	3.680	0.02561	1866	0.04315
B2	15.97	1369	3.541	0.02964	2249	0.02634
B3	18.10	2478	4.003	0.01862	5839	0.03963
B4	22.15	3415	3.967	0.01906	6252	0.03595
B5	22.73	1357	3.941	0.01974	3030	0.02853
B6	27.64	2609	3.606	0.02742	8517	0.02271
B7	29.56	4110	3.915	0.02018	6469	0.01914
C1	15.70	2411	4.316	0.01383	4240	0.05889
C2	23.44	1393	4.116	0.01688	3575	0.02810
C3	25.61	2940	4.101	0.01708	5516	0.05860
C4	28.07	1854	4.096	0.01685	6124	0.02289
C5	41.14	2393	4.268	0.01440	6347	0.03963
C6	47.69	1874	4.282	0.01425	6941	0.03754
C7	57.55	1322	4.025	0.01881	7908	0.02749
D1	12.05	639.9	4.249	0.01507	782.6	0.08514
D2	15.89	2365	4.051	0.01767	5461	0.03591
D3	28.24	2803	4.235	0.01485	4858	0.03276
D4	28.76	958.3	4.258	0.01513	2991	0.05510
D5	28.87	1387	4.146	0.01698	3901	0.04817
D6	31.85	2747	4.287	0.01396	6006	0.03268
E1	17.50	3465	4.441	0.01206	4293	0.05798
E2	19.12	1094	4.651	0.009870	1830	0.03572
E3	20.16	2698	4.504	0.01131	3811	0.02814
E4	27.76	3783	4.546	0.01073	4879	0.02180
E5	32.66	2707	4.499	0.01146	4118	0.02519
E6	33.30	3524	4.436	0.01196	4720	0.05575
E7	38.46	2236	4.442	0.01224	6014	0.009973
E8	41.48	1795	4.511	0.01226	5278	0.02993
F1	19.60	2615	4.881	0.007653	3129	0.04422
F2	20.81	862.7	4.666	0.009727	3499	0.07422
F3	23.56	2859	4.690	0.009420	3532	0.06246
F4	24.45	1532	4.778	0.008728	2764	0.05223
F5	35.13	1629	4.748	0.009311	4865	0.06013
F6	37.23	1073	4.769	0.008676	3245	0.04870
G1	12.63	1360	5.015	0.007085	1227	0.07960
G2	15.50	2900	5.204	0.005643	2442	0.08315
G3	17.37	1481	5.225	0.005581	2086	0.05848
G4	27.16	1993	5.646	0.003828	3253	0.07681
G5	27.83	3818	5.102	0.006177	3372	0.05278
G6	49.00	1172	5.127	0.006370	3836	0.02813

**Table 4:** The correlation between the heterogeneity rankings computed via various statistics and that done independently by the veteran oncologist was quantified via the Spearman rank test

					**Ranking statistic**			
**Set**	**Images**	**Area(px)**	***ζ***	**Variance**	**Entropy**	**Energy**	**Contrast**	**Homogeneity**
A	7	0–30	0.015	0.305	0.361	0.361	0.164	0.164
B	7	30–50	0.100	0.050	0.333	0.333	0.015	0.305
C	7	50–75	0.003	0.228	0.305	0.389	0.005	0.185
D	6	50–75	0.003	0.250	0.220	0.288	0.190	0.160
E	8	75–100	0.003	0.327	0.481	0.134	0.090	0.167
F	6	100-125	0.083	0.327	0.250	0.220	0.481	0.220
G	6	125–300	0.050	0.288	0.160	0.250	0.083	0.083
Mean			0.04±0.02	0.25±0.04	0.30±0.04	0.28±0.03	0.15±0.06	0.18±0.03

The *p*-values given in columns 4–9 represent the likelihood that observed correlations are due entirely to random chance.

### Critique of the method

#### Handling large images

As discussed in earlier sections, while the tumor images we study vary greatly in size relative to one another, even the largest tumor yields a fairly small number of pixel pair combinations. Thus, the isolated tumor objects are readily processed on a desktop computer. However, the computational order of *ζ* is $O(\bar{L}N^{2})$ where $\bar{L}$ is the average distance between two pixels and *N* is the number of pixels. Therefore, the exhaustive pairing we employ on the relatively small tumor regions is unlikely to be feasible on images containing a large number of object pixels. For such cases, we suggest that one take as large a random subset of all possible pixel pairings as is computationally accessible and proceed to calculate *ζ* as before.

#### Dependence upon bit depth

During computation of *ζ*, one must take the difference between a measured intensity and that predicted by the smoothest gradient. However, the smoothest gradient possible is determined by the number of shades available to transition between pixel pairs. Thus, *ζ* must depend upon image bit depth. This could be problematic when comparing image sets from different institutions which likely employ very different imaging and data storage protocols. One solution to this is the use of a common bit depth. For example, in our test images, a threshold of 40% of the maximum observed intensity was used to define the tumorous regions. Thus, only the top 60% of the grayscale range is employed to shade all intra-tumoral variations. On average, the brightest pixels corresponded to 80 kBq/mL of radioactivity (data not shown). This means that only 48 kBq/mL separate the brightest tumor regions from the dimmest. The noise associated with the FDG-PET process may be estimated from Ref. [15] to be ∼1 kBq/mL. Thus, only ≈48 shades of gray are required to shade our example objects (the tumors) in such a manner that differing shades correspond to genuinely differing amounts of radioactivity. A similar downscaling of image bit depth could be employed on a patient-by-patient basis. Statistics derived from these images (such as *ζ*) could then be meaningfully compared across patients since these variations are those common to the physics of the imaging modality, not those distinct to an arbitrary choice of bit depth.

#### Vectorization of *ζ* within one image

A directional heterogeneity quantifier is desirable for cases where the grain of the image has physical meaning. This could occur, for example, for stained histology slides [16] or for magnetic resonance images of tumors [17]. We suggest that the vectorization of *ζ* may be accomplished as follows. Again, we begin with a pair of arbitrarily labeled object pixels at coordinates (*x*_*m*_,*y*_*m*_) and (*x*_*n*_,*y*_*n*_). Instead of following the Bresenham line directly from pixel *m* to pixel *n*, one first could compute *ΔI*_*x*_ along a purely horizontal direction—say from (*x*_*m*_,*y*_*m*_) to (*x*_*n*_,*y*_*m*_)—then compute a purely vertical contribution *ΔI*_*y*_ from (*x*_*n*_,*y*_*m*_) to (*x*_*n*_,*y*_*n*_). We note that $\sqrt{{\left(\Delta I_{x}\right)}^{2}+{\left(\Delta I_{y}\right)}^{2}}$*does not equal* the *ΔI* computed directly between (*x*_*m*_,*y*_*m*_) and (*x*_*n*_,*y*_*n*_), as was described previously. For both *ΔI*_*x*_ and *ΔI*_*y*_, the ensemble-averaged, average absolute difference versus relative length curves may be constructed and corresponding *ζ* computed independently for each direction.

Consider, for example, an image consisting of uniquely shaded horizontal stripes. The directional *ζ*_*x*_=0 since, for any pixel *m*, the intensity at all horizontal distances from *m* is identical to that of *m*. That same image, however, will have a *ζ*_*y*_>0 since any vertical line must cross a stripe boundary at some distance away from *m*.

Two important caveats accompany the computation of directional heterogeneity quantifiers within an image. First, the choice of moving horizontally then vertically is completely arbitrary. As one proceeds horizontally from a given pixel, one encounters different intensity values than would be encountered had one first proceeded vertically. This ambiguity also arises when considering the order in which pixels are paired. Proceeding from the top-left of the image, the horizontal direction from *m* to *n* is defined by the upper pixel while pairing from the bottom-right of the image, it is defined by the lower pixel. Thus, even decreeing that one always proceeds horizontally first does not guarantee consistent directional *ζ*. It is for this reason that we chose to employ a purely scalar *ζ*; one which is unambiguously defined by the pixel pairs. We suggest that anyone reporting directional *ζ* also clearly report and motivate their particular procedural choices.

The second concern lies in the fact that there is no reason to presume that directional components within the image have physical meaning within the image. Therefore, what is readily computed as horizontal in the image data may not be horizontal in the real object. For example, there is no guarantee that the stripes of some striped object align with the vertical or horizontal directions of the image. Therefore, before directional quantifiers may be compared across images, some common reference frame must be implemented. We suggest that one such frame is that determined by the quadrupole moment of the image object. The origin of such a frame is given by the object dipole. The object may then be translated such that the dipole is at the center of the image then rotated such that the strongest quadrupole eigenvector aligns with (arbitrarily) the *x*-axis. This way, directional variations are measured relative to objectively determined reference frames.

#### Extension to three dimensions

We present two dimensional (2D) images test images because we were interested specifically in measuring correspondence with human experts. Two dimensional tumor images pasted onto equally-sized cards served as a ready means of presenting and manipulating the images into subjective order. Of course, an extension of *ζ* into three dimensions (3D) may be desired for a given clinical application. The Bresenham algorithm is extensible into 3D [18] and is available in many commercial software packages currently in common use. Using those 3D line data, *ζ* may be computed as described for the 2D case. A second means of extension into 3D might be as follows. Instead of drawing the Bresenham line along discrete pixels, a Euclidean line may be drawn directly to pixel pairs. The intensity value anywhere along that line may be determined by averaging and/or interpolation of the neighboring pixels. This gives *I*_*m*_ and *I*_*n*_ in Equation 1. The absolute intensity difference seen in Equation 2 may then be averaged along the Euclidean line using arbitrary intervals. That average may then be used in Equation 3 to compute *ζ* as was done in the 2D case. While we expect that a 3D *ζ* thus computed can be used to objectively rank 3D objects in order of increasing heterogeneity, the merits of one computational method over another are not clear and are a subject for further study. Additionally, if a comparison to expert opinion is to be done for 3D virtual objects, the rendering technique will also have to be scrutinized. For example, perception of 3D virtual objects varies greatly from person-to-person. Also, the construction technique—*e.g.,* brightest intensity versus average intensity along a given line of sight—adds variation to the human-computer comparison that likely requires many repeated trials to overcome. In the present work, we sought only an introduction to our automated ranking technique and purposely avoided the complications involved with extension into three dimensions.

### Applications & future work

There is increasing interest in the role of heterogeneity within the tumor microenvironment as an indicator of disease prognosis [19]. Therefore, one application is to investigate whether the *ζ* measure has any prognostic value. Cancer patients where the treatment response and/or long-term survival is known *a priori* could be ranked objectively via *ζ* heterogeneity. This could be done for some subset of two-dimensional images (such as the largest tumor cross-sections) or for three-dimensional, whole tumor, virtual objects as described above. The correspondence between heterogeneity and disease outcome could then be checked via rank testing, survival analysis or a Bayesian test of predictiveness.

Another interesting application of *ζ* lies in the association of a heterogeneity score with intra-tumoral diffusion. Diffusion tensor imaging (DTI) is a magnetic resonance modality which can measure the directional diffusion of water within a cancerous tumor [19,20]. A three dimensional tumor is imaged via two-dimensional cross sections. Each image pixel then corresponds to a three-dimensional vector representing the flow of water at the coordinate of the pixel. Unlike FDG-PET imaging—where the relative contributions of intra-cellular metabolism and inter-cellular density and arrangement are not known—the images resulting from DTI result directly from the tumor microenvironment [20]. Thus, anisotropies in the diffusion imply variations in the initiation and maintenance of tumor growth. With this in mind, it would be very interesting to investigate the relation between the bulk intra-tumoral heterogeneity and diffusion anisotropy. One means of doing this is to parse the DT images into three sets: one for each vector component of the diffusion. For inter-patient comparisons, these directions could be embedded directions relative to the tumor itself, for example, the same principal eigenvectors from which the fractional anisotropy is commonly computed [20]. The result is three sets of three-dimensional coordinates with a grayscale value at each coordinate which represents the magnitude of diffusion in a given direction at that coordinate. A three-dimensional *ζ* may be computed via Equation 3 for each set of coordinates. Those *ζ* values, each between zero and unity, may then be combined to form a single vector, $\vec{\zeta }$. This $\vec{\zeta }$ is very different from the possible vectorization discussed earlier. There, arbitrary directions were imposed upon an image to create a vector quantity within that image. Here, each image set represents diffusion measured in a distinct physical direction. Thus, $\vec{\zeta }$ is a vector where each component measures the heterogeneity of an entire three-dimensional image set, and that set measures diffusion in only one *physical* direction. If one then maps $\vec{\zeta }$ onto an RGB colorspace, the bulk heterogeneity (which could be indicative of directional diffusion) throughout the tumor bulk may then be reported as a single color. For example, it could be the case that tumors with predominately trans-axial diffusion are less treatable than those with predominately planar diffusion. These differing diffusion scenarios will yield different $\vec{\zeta }$-colors since two $\vec{\zeta }$ of the similar magnitude can still point in different colorspace directions. The $\vec{\zeta }$-color is one objective means of quantifying visually perceived image heterogeneity and relating it to a directional, physical quantity which itself feasibly relates to tumor growth. Thus, $\vec{\zeta }$ is another means of studying the relation between image heterogeneity and clinical prognosis.

## Conclusions

We have demonstrated a method for automatically ranking grayscale medical images in order of increasing heterogeneity. We have done this not only in a fashion where shape is considered to contribute to overall object heterogeneity, but also under conditions where shape is ignored. In both cases, the automatic ranking is found to agree very well with the rankings done visually by experts. The example images we analyze, specifically, those of a grayscale object against a uniformly black background, are precisely the type of images available after a clinician delineates tumors via standard image segmentation techniques. For these example cases, our shape-sensitive ranking statistic was shown to yield heterogeneity rankings which almost perfectly parallel those given by a veteran radiation oncologist. Automated ranking via heterogeneity offers a new avenue for objectively studying the clinically crucial relation between disease outcome and some tumor properties observable in the images obtained at diagnosis.

## Consent

Written informed consent for use of the data in this report was waived by the Washington University Institutional Review Board.

## Endnotes

^a^We note for clarity that the “image energy” and “local homogeneity” monikers employed by some authors actually refer to the “angular second moment” and “inverse difference moment”, respectively, as originally given in Ref. [11].^b^We note again that this is the trans-axial direction relative to the tumor—as determined by the three-dimensional principal components of the image set—which is not necessarily the trans-axial direction of the clinical imaging process.

## Competing interests

The authors declare that they have no competing interests.

## Authors’ contributions

FJB conceived and drafted the manuscript as well as performed all mathematical analyses. PWG designed the protocol for the interpretation the FDG-PET images, provided expert, independent ranking of those images, as well as created and maintained the database of clinical data and images employed herein. Both FJB and PWG read and approved the final manuscript.

## Pre-publication history

The pre-publication history for this paper can be accessed here:

http://www.biomedcentral.com/1471-2342/13/7/prepub
